# Hydrogen Nano-Bubble Water Suppresses ROS Generation, Adipogenesis, and Interleukin-6 Secretion in Hydrogen-Peroxide- or PMA-Stimulated Adipocytes and Three-Dimensional Subcutaneous Adipose Equivalents

**DOI:** 10.3390/cells10030626

**Published:** 2021-03-11

**Authors:** Li Xiao, Nobuhiko Miwa

**Affiliations:** 1 Department of Pharmacology, School of Life Dentistry at Tokyo, Nippon Dental University, Tokyo 102-8159, Japan; 2 Faculty of Life Sciences, Prefectural University of Hiroshima, Hiroshima 727-0023, Japan; jpn.cntr.antiaging.medsci2002@leto.eonet.ne.jp

**Keywords:** hydrogen-rich water, nano-bubbles, hydrogen peroxide, phorbol 12-myristate 13-acetate, IL-6, reactive oxygen species, adipogenesis, adipose equivalents, inflammation, metabolic disorders

## Abstract

Reactive oxygen species (ROS)-induced oxidative stress in adipose tissue is associated with inflammation and the development of obesity-related metabolic disorders. The aim of this study is to investigate the effects of hydrogen nano-bubble water (HW) on ROS generation, adipogenesis, and interleukin-6 (IL-6) secretion in hydrogen peroxide (H_2_O_2_) or phorbol 12-myristate 13-acetate (PMA)-stimulated OP9 adipocytes, and three-dimensional (3D) subcutaneous adipose equivalents. Nanoparticle tracking analysis showed that fresh HW contains 1.17 × 10^8^/mL of nano-sized hydrogen bubbles. Even after 8 to 13 months of storage, approximately half of the bubbles still remained in the water. CellROX^®^ staining showed that HW could diminish H_2_O_2_- or PMA-induced intracellular ROS generation in human keratinocytes HaCaT and OP9 cells. We discovered that PMA could markedly increase lipid accumulation to 180% and IL-6 secretion 2.7-fold in OP9 adipocytes. Similarly, H_2_O_2_ (5 µM) also significantly stimulated lipid accumulation in OP9 cells and the 3D adipose equivalents. HW treatment significantly repressed H_2_O_2_- or PMA-induced lipid accumulation and IL-6 secretion in OP9 adipocytes and the 3D adipose equivalents. In conclusion, HW showed a possibility of repressing oxidative stress, inflammatory response, and adipogenesis at cellular/tissue levels. It can be used for preventing the development of metabolic disorders amongst obese people.

## 1. Introduction

Obesity-induced metabolic disorders have been shown to lead to the development of type 2 diabetes mellitus and cardiovascular diseases [1,2,3]. Obesity is characterized as excessive fat accumulation in both adipose and non-adipose tissues [4]. Fat-accumulation-accompanied oxidative stress is the main contributor to the development of metabolic disorders in obesity [5,6]. Oxidative stress is caused by over-produced reactive oxygen species (ROS), which overwhelms the antioxidant defense systems in the organism [7,8]. ROS also act as intracellular redox messengers in the regulation of numerous signaling pathways ranging from cell homeostasis and cell differentiation to cell death [9]. ROS include superoxide anion (O_2_^•−^), hydroxyl radicals (•OH), hydrogen peroxide (H_2_O_2_), and hydroperoxyl radicals (HOO•). Evidence show that oxidative stress of vascular origin caused by hydrogen peroxide initiates body fat accumulation [10,11]. As the body fat expands, adipocytes and non-adipocytes (such as hepatocytes and muscle cells [12]) generate ROS. ROS-caused oxidative stress stimulates the secretion of inflammatory cytokines (such as interleukin-6 (IL-6) and tumor necrosis factor (TNF)-alpha) and adipokines, which leads to insulin resistance and other metabolic disorders [13,14,15]. Therefore, the use of antioxidants to inhibit oxidative stress might stop oxidative-stress-caused adipogenesis and the release of inflammatory cytokines and thus prevent metabolic disorders.

During the past decade, molecular hydrogen (H_2_) has gained attention as a therapeutic and preventive antioxidant by inhibiting oxidative stress in most organs [16]. Hydrogen can convert the toxic ROS, hydroxyl radicals, superoxide anion, and hydrogen peroxide to harmless water (2 •OH + H_2_ → 2H_2_O, O_2_^•−^ + 2H_2_ → 2H_2_O, H_2_O_2_ + H_2_ → 2H_2_O). It was reported that hydrogen also has anti-inflammatory effects and stimulates the energy metabolism. In this study, we tested the effects of hydrogen-bubbling water named Hexa Z Hydro-Nano-Bubble Water (HW) on hydrogen peroxide (H_2_O_2_)- or phorbol-myristate-acetate (PMA; an inducer of endogenous superoxide anion and hydrogen peroxide)-induced ROS generation, lipid accumulation, and IL-6 secretion in OP9 adipocytes and three-dimensional subcutaneous adipose equivalents.

## 2. Materials and Methods

### 2.1. Testing Hydrogen Nano-Bubbles in Hexa Z Hydro-Nano-Bubble Water

The hydrogen nano-bubble water, named Hexa Z Hydro-Nano-Bubble Water (HW; EBM Co. Ltd., Tokyo, Japan), was prepared by filtrating-extracting of the purified water through Amaterasu stone, which is mineralogically categorized as black mica-quartz obtained from Mt. Takachiho (Prefs. Miyazaki and Kagoshima, Japan), composed of SiO_2_: 66.2 (*wt*/*wt* %), Al_2_O_3_: 13.0, MgO: 4.99, Na_2_O: 4.06, Fe_2_O_3_: 2.95, CaO: 2.45, K_2_O: 1.10, and TiO_2_: 0.44, with far-infrared radiation of >86.3% and atmospheric negative ion emission of >35 ions/mL, thereafter bubbling through high-porosity microporous terminals with hydrogen gas under high pressure, and sterilizing with a filter. HW was packed in screw-capped aluminum bottles and preserved below room temperature. A possibility for formation and existence of water cyclic-hexamer clusters was characterized IR-spectroscopically by Nakamura and Ito [17]. Nano-sized hydrogen bubbles in HW were analyzed using Nanoparticle Tracking Analysis (NTA; Version 2.3 Build 0025, Malvern, UK) with a Nano Sight LM10V-HS system (Malvern Co. Ltd., Malvern, UK). The water samples obtained at different time points were stored at 4 °C until use. The storage times were 8 months, 13 months, 15 months, and 8 years. Polystyrene latex particles with a diameter of 100 nm in water were used as standard particles. Each sample was tested 6 times.

### 2.2. Cell Culture

Human immortalized skin epidermal keratinocytes (HaCaT) were kindly provided by Professor Norbert E. Fusenig of the Deutsches Krebsforschungszentrum (Heidelberg, Germany) [18]. Cells were maintained in Dulbecco’s modified Eagle’s medium (DMEM; Thermo Fisher Scientific, Tokyo, Japan) supplemented with 10% fetal bovine serum (FBS), 1% Gibco^®^ GlutaMAX™ Supplement (Thermo Fisher Scientific, Tokyo, Japan), 100 units/mL penicillin, and 10 mg/mL streptomycin in a 5% CO_2_ atmosphere at 37 °C [19].

OP9 mouse stromal cells (RCB1124) were purchased from the Riken Cell Bank (Saitama, Japan). Cells were cultivated in the propagation medium: MEM-α with 20% FBS, 1% Gibco^®^ GlutaMAX™ Supplement (Thermo Fisher Scientific), 100 units/mL penicillin, and 10 mg/mL streptomycin in a 5% CO_2_ atmosphere at 37 °C. For adipocyte differentiation, OP9 cells were grown to 100% confluence in the propagation medium. Cells were then cultivated in KSR medium (MEM-α containing 15% KnockOut™ serum replacement, 100 units/mL penicillin, 10 mg/mL streptomycin, and 1% Gibco^®^ GlutaMAX™ Supplement) for another 4–6 days [20].

### 2.3. D Culture of Subcutaneous Adipose Equivalents

The subcutaneous adipose equivalents were prepared with a Cellmatrix type I-A culture kit (Nitta Gelatin, Tokyo, Japan) similar to our previous study [20]. Briefly, OP9 cells (1 × 10^6^ cells/mL) were gently added into the type I-A collagen gel mixed with 10% 10× concentrated MEM- α and 10% reconstruction buffer (2.2 g of NaHCO_3_ and 4.47 g HEPES in 100 mL 0.05 N NaOH). Then, the mixture was seeded into a Falcon^®^ 24-well culture insert (Corning Inc., Tokyo, Japan) and placed into a 24-well plate. After the mixture was incubated in OP9 cells propagation medium for 3–4 days, HaCaT cells (1 × 10^6^ cells/mL) were seeded into the OP9 collagen gel substrate and the medium was changed to KSR medium containing 5% FBS. One day later, the medium was replaced with KSR medium containing 1% FBS. After further cultivation for 4 days, the culture surfaces were exposed to air in the incubator. The culture medium was replaced with KSR medium without FBS twice a week and the cells incubated for another 2–4 weeks.

### 2.4. Hydrogen Peroxide and PMA Treatment

Hydrogen peroxide (H_2_O_2_ 30%; 081-04215, FUJIFILM Wako Pure Chemical Co., Osaka, Japan) and PMA (162-23591; FUJIFILM Wako Pure Chemical Co., Osaka, Japan) were first diluted in double-distilled water (DDW), and then in culture medium just prior to each experiment. For hydrogen peroxide treatment, HaCaT or OP9 cells were incubated in culture medium with H_2_O_2_ at different concentrations for certain culture periods. Control cells were treated similarly without H_2_O_2_. For PMA treatment, OP9 cells were incubated with PMA (10 ng/mL) in KSR medium for 4 days. Control cells were similarly treated without PMA.

### 2.5. Live/Dead Staining

Live and dead cells were detected with a Live/Dead^®^ Cell Imaging kit (488/570; R37601; Thermo Fisher Scientific) according to the manufacturer’s protocol. The images were observed with a confocal laser scanning microscope (LSM 700; Carl Zeiss Microscopy Co., Ltd., Tokyo, Japan).

### 2.6. Cell Viability Assay

Cell viability in HaCaT cells was measured using PrestoBlue^®^ Assay according to the manufacturer’s protocol. At the end of cultivation, HaCaT cells were incubated for 3 h at 37 °C in fresh medium supplemented with 10% PrestoBlue^®^ (*v*/*v*; A13261, Thermo Fisher Scientific). The PrestoBlue^®^ reduction, expressed as fluorescence intensity units by the cells, was measured using a microplate reader (SH-9000Lab, HITACHI, Tokyo, Japan) with excitation/emission at 560 nm/590 nm [21].

### 2.7. Cellular ROS Detection

HaCaT cells were cultivated in DDW- or HW-prepared culture medium for 2 h. The medium was then changed to regular medium containing H_2_O_2_ at different concentrations. At 2 h after H_2_O_2_ treatment, cellular ROS generation in HaCaT cells was detected using the CellROX^®^ Orange or Green Reagent (Thermo Fisher Scientific) according to the manufacturer’s recommended protocol. ROS production in cells was observed by the EVOS^®^ FL Cell Imaging System (Thermo Fisher Scientific). To qualitatively analyze the ROS production, HaCaT cells were detached from the culture substratum-surface using a 0.25 *w*/*v*% trypsin, 1 mmol/L EDTA solution, and then suspended in PBS (−) at a concentration of 1 × 10^6^ cells/mL. 25 µL of the cell suspension was infused into a Tali™ Cellular Analysis Slide (T10794, Thermo Fisher Scientific) and analyzed by the Tali™ Image Cytometer (Thermo Fisher Scientific) [19].

### 2.8. Oil Red O Staining

Specimens were fixed with 10% formalin and then stained for 1 h with filtered solution of 0.25% Oil Red O in 60% aqueous 2-isopropanol. After being washed with distilled water, samples were further stained with hematoxylin for 10 min. The images of stained samples were observed with a light microscope (Nikon, Tokyo, Japan). For quantitative analysis of cellular lipid droplets in OP9 cells, hematoxylin staining was omitted. Cellular dye was extracted with 60% isopropanol using a platform rocker for 30 min. The extracted dye was transferred into a 96-well plate and read for absorbance with a microplate reader (SH-9000Lab, HITACHI) at 530 nm [22]. For quantitative analysis of cellular lipid droplets in 3D subcutaneous adipose equivalents, the images of Oli Red O staining were analyzed using ImageJ software (v1.52, National Institute of Health, Bethesda, MD, USA).

### 2.9. Enzyme-Linked ImmunoSorbent Assay (ELISA)

To determine levels of IL-6 in OP9 cells, samples were analyzed using a commercially available mouse IL-6 ELISA Kit, (RAB0308, Sigma-Aldrich, Tokyo, Japan). At the end of the cultivation process, cell culture supernatants were collected and reacted with the first and secondary antibodies, streptavidin-HRP, and detection solution according to the manufacture’s instruction. The reaction was stopped using the stop solution (from the ELISA kits) and absorbance was read at 450 nm using a microplate reader (SH-9000Lab, Hitachi, Tokyo, Japan) [23].

### 2.10. Statistical Analysis

Statistical analysis was carried out similarly to our previous report [24]. All data, expressed as the mean ± SD, were analyzed statistically using GNU PSPP Statistical Analysis Software (version 0.8.2-gad9374; (https://www.gnu.org/software/pspp/) and EZAnalyze Excel-based tools (http://www.ezanalyze.com/). A one-way analysis of the variance was followed by post hoc tests (including Tukey’s test and Bonferroni correction). Statistical significance was considered when *p* < 0.05. All experiments were repeated 3–5 time, independently.

## 3. Results

### 3.1. HW Contains 100 Million Fine Hydrogen Bubbles

**Figure 1:**
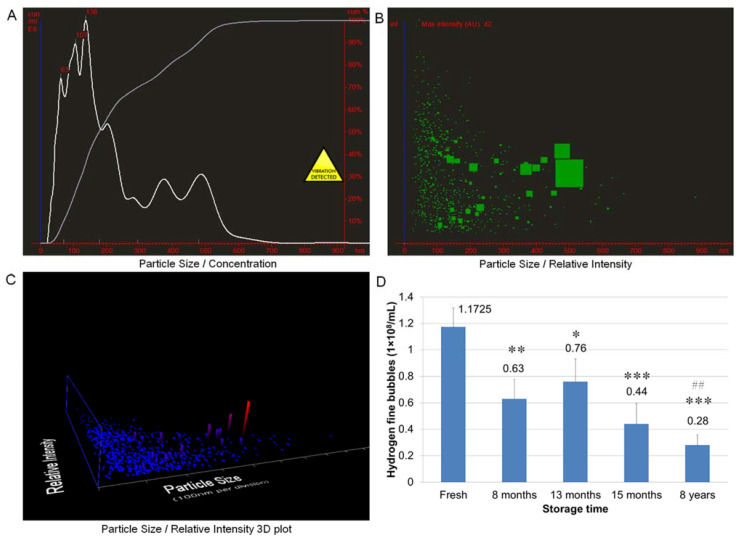
Concentration of hydrogen nano-bubbles in hydrogen nano-bubble water (HW). Nano-sized hydrogen bubbles in HW with different storage times were analyzed using Nanoparticle Tracking Analysis (NTA) software. Typical analysis results of particle size, distribution and number in HW are presented in (**A**–**C**). Numbers of hydrogen fine bubbles in HW samples were shown in (**D**). * *p* < 0.05, ** *p* < 0.01, *** *p* < 0.001 vs. fresh, ## *p* < 0.01 vs. 8 months. Each bar represents the mean ± SD of six independent experiments.

The dissolved hydrogen escapes from the water very easily. We demonstrated that the half-life-time of dissolved hydrogen in regular hydrogen water was about 40 min. However, if the hydrogen molecules were wrapped in nano-sized fine bubbles, the hydrogen did not easily escape. The NTA analysis showed that there were over 100 million fine bubbles in fresh HW. The average size of the bubbles was about 100 nm (Figure 1A–C). The number of bubbles decreased to approximately half after 8 months under the conditions in a screw-capped aluminum container with no air exchange. However, there was no significant difference between the 8, 13, and 15 month old HW. Surprisingly, after 8 years of storage time, 24% of the hydrogen bubbles still remained in the HW. These data suggest that hydrogen nano-bubbles in HW are very stable during storage.

### 3.2. Repressive Effects of HW on H_2_O_2_-Induced Cell Death in HaCaT Cells

**Figure 2:**
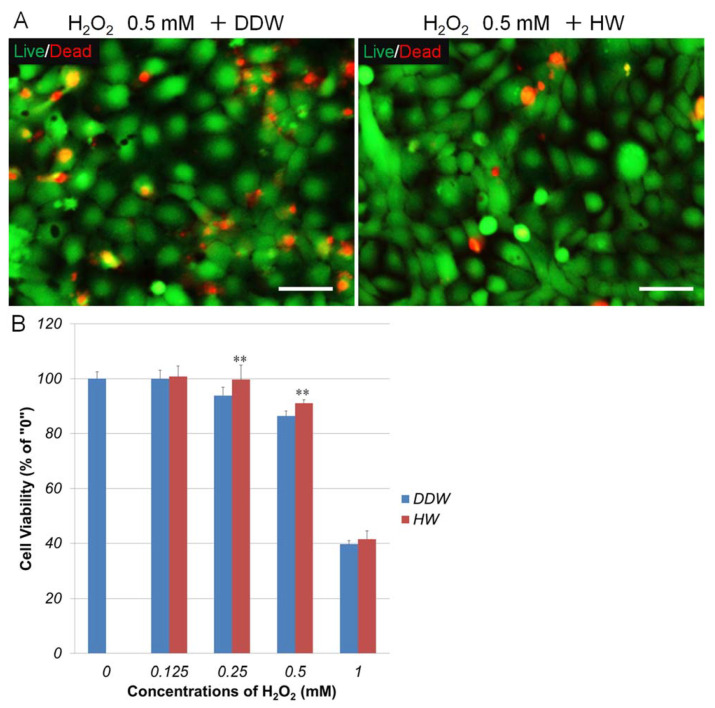
Repressive effects of HW on hydrogen peroxide-induced cell death in human keratinocytes HaCaT. HaCaT cells were pretreated with double-distilled water (DDW)- or HW-prepared medium for 2 h. Cells were then exposed to H_2_O_2_ at different concentrations. After 48 h, cell viability was measured by PrestoBlue Assay. (**A**) HaCaT cells were stained by Live/Dead staining. Green, live cells; red, dead cells. Scale bar = 50 µm (**B**) results of PrestoBlue Assay. ** *p* < 0.01 vs. 0 (without H_2_O_2_ treatment). Each bar represents the mean ± SD of three independent experiments.

We tested the effects of HW on H_2_O_2_-induced cell death in HaCaT cells using two methods: live/dead staining and the PrestoBlue Assay. As shown in Figure 2B, H_2_O_2_ decreased cell viability in HaCaT cells in a concentration-dependent manner. HW showed significant protective effects in 0.25 and 0.5 mM H_2_O_2_-treated cells. Live/dead staining confirmed that HW reduced dead cells in 0.5 mM H_2_O_2_-treated cells. These data suggest that HW could protect HaCaT cells from H_2_O_2_-induced damage.

### 3.3. Repressive Effects of HW on H_2_O_2_-Induced Cellular ROS Generation in HaCaT Keratinocytes

**Figure 3:**
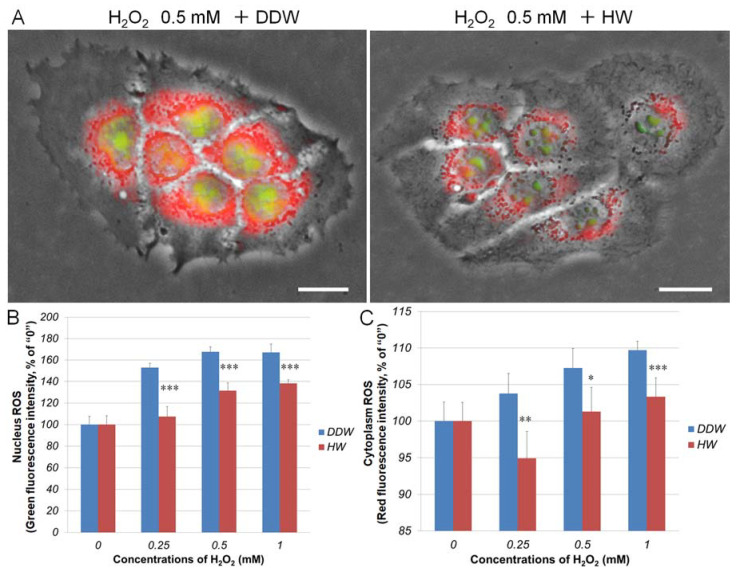
Repressive effects of HW on hydrogen peroxide-induced cellular ROS in human keratinocytes. HaCaT cells were pretreated with DDW- or HW-prepared medium for 2 h. Cells were then exposed with H_2_O_2_ at different concentrations. At 24 h after H_2_O_2_-treatment, cellular ROS was detected with CellROX^®^ green and orange dyes and observed with a fluorescence microscope. To quantitatively analyze the cellular ROS, green and red fluorescence in HaCaT cells were measured with the Tali image-based cytometer. (**A**) Typical images of CellROX^®^ green and orange staining. Green, nucleus ROS; red, cytoplasm ROS. Scale bar = 10 µm. (**B**) and (**C**) HaCaT cells were detached from the culture substratum-surface by trypsin and followed with Tali-cytometric analysis to determine nucleus and cytoplasm ROS as described in materials and methods. * *p* < 0.05; ** *p* < 0.01; *** *p* < 0.01 vs. 0 (without H_2_O_2_ treatment). Each bar represents the mean ± SD of three independent experiments.

As shown in Figure 3, H_2_O_2_ treatment caused ROS generation in both the cytoplasm and nuclei in HaCaT cells. Cellular ROS production was increased in H_2_O_2_-concentration-dependent manner. When HaCaT cells were pre-cultivated in HW-prepared medium for 2 h, both the nuclei and cytoplasm ROS were significantly reduced, suggesting that HW could scavenge cellular ROS.

### 3.4. Effect of HW on H_2_O_2_-Stimulated Adipogenesis in KSR-Differentiated OP9 Adipocytes and 3D Subcutaneous Adipose Equivalents

**Figure 4:**
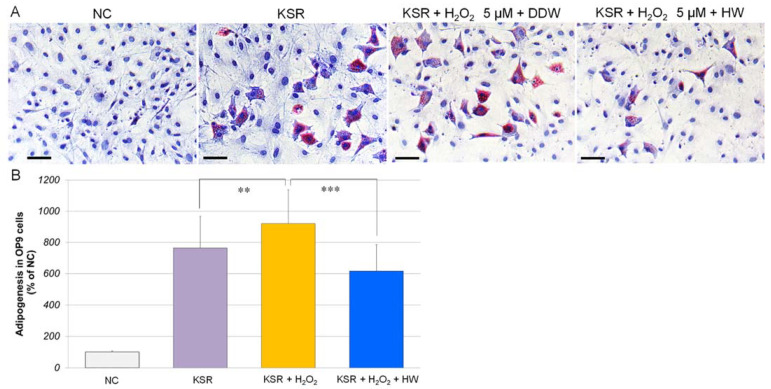
Repressive effects of HW on hydrogen-peroxide-stimulated adipogenesis in OP9 cells. OP9 preadipocytes (30,000 cells/well) were seeded into 24-well plates and grew to 100% confluence in the maintenance medium. Cells were then cultivated in DDW- or HW-prepared KSR medium with or without H_2_O_2_ for 4 days. The negative control (NC) cells were cultivated in the maintenance medium. At the end of cultivation, cells were fixed with 10% formalin. Cellular lipid droplets were stained with Oil Red O dye. The intracellular dye was extracted and transferred to a 96-well plate, and quantified in a microplate reader at 520 nm. (**A**) Typical images of Oil Red O staining. Blue, nuclei; red, lipid droplets. Scale bar = 25 µm. (**B**) Data are expressed as a percentage of the negative control, and each concentration point and bar represent the mean ± SD of five independent experiments. ** *p* < 0.01; *** *p* < 0.001.

In our previous study, we demonstrated that H_2_O_2_ at a low concentration (5 µM) could stimulate adipogenesis in 3T3-L1 preadipocytes [20]. In this study, we found that H_2_O_2_ (5 µM) also significantly increased adipogenesis to 120% in KSR-differentiated OP9 adipocytes (Figure 4). Figure 4A shows that after being cultivated in KSR medium for 4 days, OP9 cells accumulated lipid droplets, especially in H_2_O_2_-treated cells. However, when OP9 cells were cultivated in HW-prepared KSR medium, the lipid accumulation significantly reduced.

**Figure 5:**
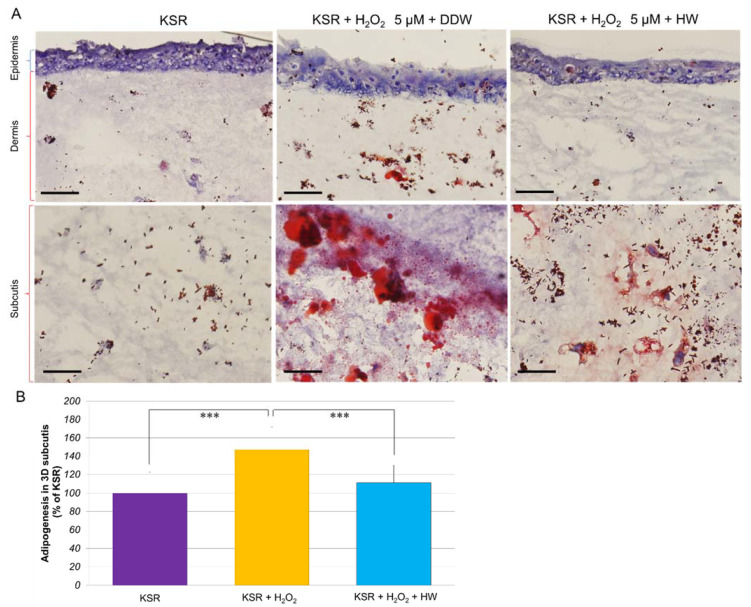
Repressive effects of HW on hydrogen peroxide-stimulated adipogenesis in 3D subcutaneous adipose equivalents. 3D equivalents were constructed as described in Materials and Methods. The 3D equivalents were cultivated in DDW- or HW-prepared KSR medium with or without H_2_O_2_ (5 µM) for 4 days. At the end of cultivation, the tissue was fixed with 10% formalin and subjected to the frozen section. The lipid droplets were stained with Oil Red O dye. The images were taken by a microscope and analyzed with ImageJ software. (**A**) Typical images of Oil Red O staining. Blue, nuclei; red, lipid droplets. The scale bars indicate 100 µm in the upper panel and 50 µm in the lower panel, respectively; (**B**) data are expressed as a percent of KSR, and each concentration point and bar represent the mean ± SD of five independent experiments. *** *p* < 0.001.

We further examined whether HW had similar effects on H_2_O_2_-stimulated adipogenesis in 3D subcutaneous adipose equivalents. Figure 5 shows that with H_2_O_2_ (5 µM) treatment, there were about 145% more lipid droplets accumulated in the subcutis of KSR-differentiated adipose equivalents. When the equivalents were cultivated in HW-prepared KSR medium, the lipid accumulation was significantly reduced to 110%.

These data suggest that HW could suppress H_2_O_2_ adipogenesis in both OP9 adipocytes and 3D adipose equivalents.

### 3.5. Effect of HW on PMA-Induced ROS Generation, Adipogenesis, and IL-6 Secretion in KSR-Differentiated OP9 Adipocytes

**Figure 6:**
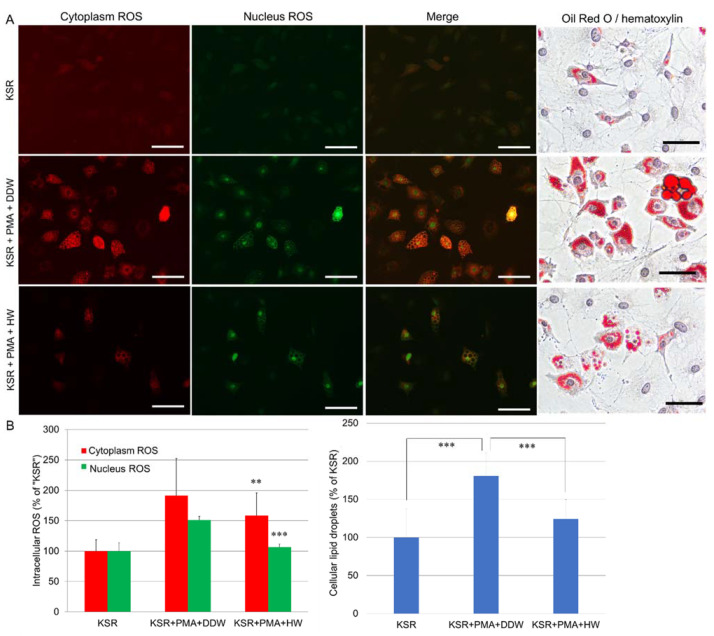
Repressive effects of HW on phorbol 12-myristate 13-acetate (PMA)-induced intracellular ROS and lipid accumulation in OP9 adipocytes. (**A**) OP9 preadipocytes (30,000 cells/well) were seeded into 24-well plates and grew up to 100% confluence in the maintenance medium. Cells were then cultivated in DDW- or HW-prepared KSR medium with or without PMA of 10 ng/mL for 4 days. The NC cells were cultivated in the maintenance medium. At the end of cultivation, cellular ROS were detected with CellROX^®^ green and orange dyes and observed with a fluorescence microscope. To quantitatively analyze the cellular ROS, green and red fluorescence in OP9 cells were measured with the Tali image-based cytometer. For Oil Red O staining, OP9 cells were prepared similar to what we described in Figure 4. (**A**) Left panel:typical images of CellROX^®^ green and orange staining. Green, nucleus ROS; red, cytoplasm ROS. Scale bar = 25 µm. Right panel: typical images of Oil Red O staining. Scale bar = 25 µm. (**B**) Left panel: OP9 cells were detached from the culture substratum-surface by trypsin and followed by Tali-cytometric analysis as described in materials and methods. ** *p* < 0.01; *** *p* < 0.01 vs. KSR+PMA+DDW. Right panel: after Oil Red O staining, the intracellular dye was extracted and transferred to a 96-well plate and quantified in a microplate reader at 520 nm. *** *p* < 0.001 vs. KSR+PMA+DDW. Data are expressed as percentage of the negative control, and each concentration point and bar represent the mean ± SD of five independent experiments.

PMA was used as a NADPH oxidase activator, which induces endogenous superoxide anion and hydrogen peroxide in macrophages [25]. PMA also induces ROS generation and oxidative stress in human leukemia cell lines: THP-1 and U937 cells [26,27]. Both THP-1 and U937 cells can be differentiated into macrophage-like cells after being treated with PMA [28,29]. In this study, we demonstrated for the first time that PMA could increase lipid accumulation in KSR-differentiated OP9 cells. As shown in Figure 6, after being cultivated in KSR medium with PMA (10 ng/mL) for 4 days, cellular lipid droplets increased by 180% compared with the cells treated with KSR alone. At the same time, the ROS level of both cytoplasm and nuclei in OP9 cells was also markedly increased. When OP9 cells were cultivated in HW-prepared KSR medium with PMA for 4 days, cellular lipid droplets, nuclei, and cytoplasm ROS were significantly reduced, suggesting that HW was able to inhibit PMA-induced intracellular ROS and lipid accumulation.

**Figure 7:**
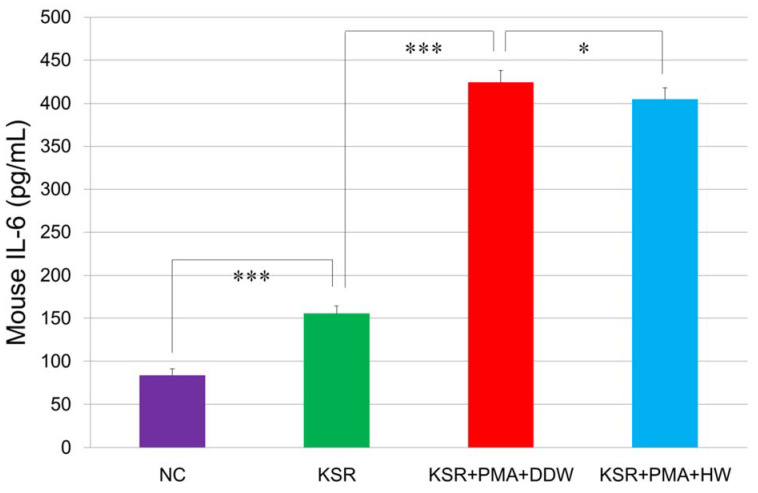
Repressive effects of HW on PMA-increased IL-6 secretion in OP9 adipocytes. OP9 preadipocytes (30,000 cells/well) were seeded into 24-well plates and grew to 100% confluence in the maintenance medium. Cells were then cultivated in DDW- or HW-prepared KSR medium with or without PMA of 10 ng/mL for 4 days. The NC cells were cultivated in the maintenance medium. At the end of cultivation, concentrations of IL-6 in cell culture supernatant were measured by ELISA as described in Materials and Methods. * *p* < 0.05; *** *p* < 0.001. Each bar represents the mean ± SD of three independent experiments.

The inflammatory cytokine IL-6 is known to play an essential role in the inflammation of adipose tissue and insulin resistance [15]. Figure 7 shows that during KSR-initiated differentiation OP9 adipocytes secreted 1.8 times more IL-6 than the negative control. PMA-treatment further increased the level of IL-6 by 270% of KSR-differentiated adipocytes, suggesting that PMA could markedly stimulate the secretion of IL-6 in OP9 adipocytes. However, when OP9 cells were cultivated in HW-prepared KSR medium with PMA for 4 days, IL-6 were significantly decreased, suggesting that HW was able to inhibit PMA-induced IL-6 secretion.

## 4. Discussion

Hydrogen molecules can easily permeate cellular membranes and reduce oxidative stress [30]. Recently, a randomized, double-blind, controlled clinical trial reported that hydrogen-rich water could increase serum antioxidant capacity and reduce inflammatory responses in healthy adults [31]. However, dissolved hydrogen molecules escape very easily from the solution. Our previous study showed that the half-time of dissolved hydrogen in magnesium stick-prepared hydrogen-rich water was only 40 min [19]. Fine bubble technology is a newly established technology that has been developed in medical, pharmaceutical, and dental applications in Japan. Fine bubbles in liquids exhibit excellent stability and permeability. In the present study, we demonstrated that fine hydrogen nano-bubbles are very stable in water. The number of hydrogen nano-bubbles remained in HW for a long period of storage time. Even after 8 years of storage, 24% of the bubbles remained.

Our cellular experiments showed that H_2_O_2_ at higher concentrations (0.25–1 mM) severely damaged HaCaT keratinocytes, resulting in cell death and the production of ROS. HW could inhibit H_2_O_2_-induced ROS generation; therefore, prevent H_2_O_2_-caused cell death in HaCaT cells. As one of the harmful ROS, H_2_O_2_ facilitates adipocyte differentiation [32]. It was reported that H_2_O_2_ at a concentration as low as 5 µM did not show cytotoxicity [33]. Our data showed that H_2_O_2_ at 5 µM could increase the cellular lipid accumulation to 120% of KSR-differentiated OP9 adipocytes. It was reported that subcutaneous adipose tissue (SAT) releases IL-6 in vivo [34]. Expanded SAT increases the metabolic and cardiovascular risk in some populations [35]. Here, we reconstructed 3D subcutaneous adipose equivalents using HaCaT keratinocytes and OP9 preadipocytes. As shown in Figure 5, after being cultivated in KSR medium for 2–4 weeks, HaCaT cells formed an epidermis with multilayers. Under the epidermis, OP9 preadipocytes formed dermis with only a few lipid droplets. Beneath the dermis, OP9 cells differentiated into adipocytes accompanying massive lipid accumulation, especially with H_2_O_2_ stimulation, indicating the formation of subcutis. HW significantly inhibited H_2_O_2_-induced adipogenesis in both KSR-differentiated OP9 adipocytes and 3D subcutaneous adipose equivalents.

The ROS initiator, PMA, is also known as a potent tumor promoter that activates the signal transduction enzyme protein kinase C (PKC) and differentiates cancerous lymphocytes, such as THP-1 cells [36,37]. Since PKC signaling activates inflammatory responses, PMA is often used as a potent inducer of inflammation in animal models [38,39]. In our previous study, we found that 10 ng/mL of PMA could efficiently induce the differentiation of THP-1 cells, as did other researchers [40,41,42]. We also discovered that a longer duration of treatment (more than 4 days) with PMA can promote maturation of THP-1 macrophages (data not shown). In this study, we discovered for the first time that PMA (10 ng/mL, 4 days) could enhance adipogenesis by 180% and IL-6 secretion as abundant as 270% of KSR-differentiated OP9 adipocytes. PMA also increased cellular ROS generation in OP9 adipocytes. This discovery clarifies the relationship among inflammation, adipogenesis, and oxidative stress in obesity at the cellular level in vitro. PMA-stimulated OP9 adipocytes can be applied as a new cell model for metabolic disorders. HW significantly suppressed PMA-induced lipid accumulation, ROS generation, and IL-6 secretion in OP9 cells, suggesting it might prevent the development of metabolic disorders. Since oxidative stress and inflammation play essential roles in many chronic metabolic diseases and hydrogen water is known for lacking adverse effects [30], the use of hydrogen water can be applied for the prevention of these diseases. However, more randomized, double-blind, controlled clinical trials and well-designed long-term cohort studies need to be performed in the future.

## 5. Conclusions

In conclusion, HW exhibits excellent stability and can protect HaCaT cells from harmful H_2_O_2_-induced injuries. HW significantly suppressed H_2_O_2_- or PMA-enhanced adipogenesis, ROS generation, and IL-6 secretion in OP9 adipocytes and 3D subcutaneous adipose tissue. Therefore, HW can be applied as a potential health care candidate for preventing metabolic disorders.

## Data Availability

The data presented in this study are available within the article. There are no databases associated with this manuscript.

## Abbreviations

HW	Hexa Z Hydro-Nano-Bubble Water
DDW	Double-distilled water
PMA	Phorbol 12-myristate 13-acetate
IL-6	Interleukin-6
ROS	Reactive oxygen species
3D	Three-dimensional
KSR	KnockOut™ serum replacement
NTA	Nanoparticle Tracking Analysis
